# Optimization path of primary public health service talent team construction: a largescale survey in Huaihai Economic Zone, China

**DOI:** 10.3389/fpubh.2024.1399857

**Published:** 2024-08-21

**Authors:** Yuting Ni, Yan Wang, Zongliang Wen, Jinhua Fang, Jintao Xu, Shenqin Wu, Joyce D. Sawmadal, Hamdi Abdirizak Jama

**Affiliations:** ^1^School of Management, Xuzhou Medical University, Xuzhou, China; ^2^Affiliated Hospital of Xuzhou Medical University, Xuzhou, China

**Keywords:** burnout, team construction, Huaihai Economic Zone, primary public health service, work–family conflict

## Abstract

**Background:**

The primary public health service system is indispensable for the implementation of the “Healthy China 2030” strategy, and primary healthcare workers, as the key drivers of this system, play a pivotal role in its development and establishment to ensure population well-being. In developing countries, such as China, primary public health systems are still weak, and in order to address this phenomenon, health system reform is needed, and primary public health personnel are crucial to health system reform. The current situation of primary public health workers in low-income and developing countries is characterized by varying degrees of problems that need improvement.

**Objectives:**

The purpose of this study is to understand the current situation of primary public health service workforce building, analyze the existing problems of the workforce, put forward suggestions for improvement and explore countermeasures, and provide Chinese wisdom and a reference basis for primary public health workforce building in the world, especially in developing countries.

**Methods:**

Combining the Work–Family Conflict Scale, Copenhagen Burnout Inventory, Minnesota Satisfaction Questionnaire, and Turnover Intention Scale, a relevant survey questionnaire was designed to quantitatively investigate the baseline characteristics of primary public health service institutions and their staff in four representative cities in the Huaihai Economic Zone: Xuzhou in Jiangsu Province, Linyi in Shandong Province, Shangqiu in Henan Province, and Huaibei in Anhui Province. The collected data were analyzed and processed using SPSS 25.0 statistical analysis software through univariate analysis and logistic regression analyses. Methods such as one-way ANOVA, Logistic regression analysis, and independent samples *t*-test were used to analyze the influencing factors of primary public health workforce development.

**Results:**

The current work intensity at the primary public health level is currently high, the salary and benefits cannot meet the needs of most primary public health personnel, and the competition between work and family in terms of time and resources is pronounced, and the majority of primary public health personnel are dissatisfied with the status quo of “doing more work for less reward” and the poor social security. Emotional exhaustion, depersonalization, and a sense of personal accomplishment were positively correlated with the tendency to leave (all *p* < 0.01), and the burnout and emotional exhaustion of primary public health workers were intense.

**Conclusion:**

Primary public health personnel play an important role in providing primary public health services. However, the current working conditions of junior public health personnel in the Huaihai Economic Zone are influenced by factors such as workload, income level, and employment situation improvement, leading to low job satisfaction, significant work–family conflicts, and high turnover intention. In this context, based on the opinions of grassroots administrative departments and internationally relevant experiences, a series of suggestions have been proposed to improve the professional service level, job satisfaction, and occupational identity of staff members. These suggestions make valuable contributions to both the Huaihai Economic Zone and countries worldwide in safeguarding individual health and promoting national primary healthcare reform.

## 1 Introduction

As an essential pillar in ensuring people’s health, primary public health services are indispensable in the process of promoting the “Healthy China 2030” strategy. The talent pool of primary public health service professionals serves as the main force behind the development and improvement of the primary public health service system, playing a crucial role (1). Developing countries, such as China, still need more vital health systems, and with the rapid increase in the cost of disease, they will not be able to sustain their response to this enormous disease burden (2). Responding to this phenomenon requires reform of the health system, which can only function if it is staffed with health workers.

Since 2016, 66 Member States have reported data on the stock of community health workers on the World Health National Manpower Accounts data platform, totaling 2.4 million based on the latest available data reported by these Member States (3). According to WHO, by 2030, there will be a projected global shortage of 18 million health workers, mainly in low-income and developing countries. However, countries at different levels of socio-economic development need help in educating, employing, deploying, retaining, and performing their workforce.

It is clear that promoting the development of primary organizations and strengthening the capacity of health services is of paramount importance in building a primary public healthcare system. Cultivating multidisciplinary and complex human resources with a solid foundation of specialized knowledge, skilled health practice, and professional competence in primary public health services is an issue that needs to be addressed urgently (4).

Many developed countries have formed a more mature system of training personnel for primary public health services, which plays a vital role in guiding the training and practice of primary public health personnel. For instance, in Germany, those who aspire to practice medicine must pass three national examinations to assume the position of an assistant doctor. After completing a 5-6-year specialized training in a hospital and passing the qualifying examination for specialized doctors, they can become formally, contracted doctors or establish their clinics. The majority of the population in the United States enjoys comprehensive, coordinated, continuous, follow-up, and accountable primary healthcare services. The primary healthcare system in the United States mainly comprises family doctors, general internists, general pediatricians, and geriatricians who have undergone a three-year residency training after graduating from medical school (5). Some surveys have shown that organizational communication and job satisfaction are key factors influencing healthcare professionals to practice in healthcare facilities in countries under pressure from high unemployment, economic constraints, and staff shortages. In contrast, in areas with less developed infrastructure, the satisfaction of primary public health care workers is mainly influenced by their managers (6). Soonman studied the turnover of primary public health workers and found that 64% of those surveyed were not treated with the respect they deserved, that there were limitations in the promotion pathways in primary health care, and that the overall social identity of the practitioners was also a factor affecting job satisfaction (7). Jankelová believes that a good hospital environment and infrastructure can increase the satisfaction of primary healthcare workers, and Sharifa found that optimizing health policies, setting reasonable working hours, and improving remuneration can increase the sense of belonging and satisfaction and slow down the brain drain (8).

Unbalanced allocation of social and health human resources, primary health care institutions, and rural areas need to be more talented people. Such problems become the main obstacles to the construction of the health workforce (9). Greiner believes that it is necessary to publicize and educate students of medical schools in higher education institutions and specialists in health schools to encourage them to go to primary health care institutions to improve the problem of the scarcity of public health personnel at the grassroots level (10). In his study, Niskala said there is a need to strictly control the human resources of primary health care organizations and attach great importance to the cultivation and improvement of the motivation of primary health care personnel (11). Tyson also mentioned in his study that it is necessary to adopt diversified communication methods and provide relevant training for primary health care workers to continuously improve primary health care services and promote the solution of chronic diseases at the grassroots level (12). Aytona et al. (13) used a multi-stage sampling method to provide reference suggestions for shifting the decision-making of primary public health personnel construction from the perspective of staffing and other perspectives. Therefore, it is vital to provide adequate resources for the talent building through the combined efforts of multiple organizations.

Yajun et al. used various methods, such as census and Kruskal-Wallis H test, to investigate the burnout situation of primary public health workers in Wanzhou District and a county in Zhejiang Province to adjust the service compensation system of primary public health service workers (14, 15). Shichao et al. (16) used a multi-stage sampling method and convenience sampling method to draw samples, adopted a semi-structured interview method to study the primary public health service personnel, and analyzed the information obtained from the research on burnout with the help of multivariate hierarchical linear regression, Pearson analysis, and thematic framework method. Therefore, analyzing the current situation of burnout of primary public health personnel from multiple perspectives and in multiple ways is the only way to more effectively and accurately take the pulse of the factors that genuinely affect the flow of primary public health personnel.

In order to focus on building a medical service center in the Huaihai Economic Zone, consolidate and improve the supply capacity of regional medical services, improve the regional medical cooperation mechanism, and realize the downward transfer of high-quality medical resources so that more people can enjoy high-quality medical resources. Based on the consideration of economic, political, geographic, and cultural background reasons, this study chooses four cities, Xuzhou, Linyi, Shangqiu, and Huaibei, as representatives of the Huaihai Economic Zone, and conducts an in-depth investigation to analyze the current situation of the construction of primary public health care personnel in the four cities. On this basis, we will try to overcome the existing development bottlenecks and alleviate the difficulties in building primary public health care personnel by combining international experiences to share our experiences with low-income and middle-income countries in both theory and reality and improve the national health level (17).

## 2 Materials and method

### 2.1 Participants, procedure, and ethics statement

The information for this study is based on data from extensive multi-stage online and offline research on the current status of health service personnel in primary public health organizations in four cities in the Huaihai Economic Zone.

The Huaihai Economic Zone, comprising 14 prefecture-level cities in Jiangsu, Anhui, Henan, and Shandong provinces, is one of China’s earliest regional economic cooperation organizations. Geographically located at the eastern fortress of the Eurasian Continental Bridge, it serves as a vital bridge connecting China’s three major economic belts: East, Central, and West. With its southern border adjacent to the Yangtze River Delta and its northern connection to the Bohai Rim Economic Zone, this core area covers a population of 120 million people and faces significant healthcare service challenges. Amongst the top 5 cities within the Huaihai Economic Zone, Xuzhou City plays a central role in leading economic development within the region similar to Shanghai’s position in the Yangtze River Delta. Linyi City holds strategic importance for Shandong Province while also being renowned as a revolutionary base with abundant cultural resources. Shangqiu City ranks among Henan Province’s forefronts economically with a GDP surpassing 300 billion yuan in 2021 while possessing a rich cultural heritage. Huai’bei City represents one of Anhui Province’s middle-to-lower reaches as an epitome of Huai River culture despite experiencing slower economic development.

Considering these contextual factors, Xuzhou, Linyi, Shangqiu, and Huai’bei have been chosen as representative research sites within the Huaihai Economic Zone. In-depth investigations will be conducted to analyze their current status regarding grassroots public health personnel construction while providing targeted recommendations to support further development in this aspect.

Based on the consideration of regional geographic location and economic level, the research was divided into two stages. The first stage is based on on-site research, using stratified random whole cluster sampling to select four primary hospitals in Xuzhou City as a pilot for on-site investigation. At the same time, combined with interviews, doctors were interviewed to understand their workflow, and on-site data collection was carried out to understand further the current situation and dilemmas of primary public health personnel practicing medicine and give the team a direction for multi-dimensional thinking.

The second stage was based on online questionnaires. The questionnaires of the pre-study surveyed the personal job information, job satisfaction, job intensity, and job willingness of primary public health personnel in six main urban areas (Yunlong District, Jiawang District, Tongshan District, Gulou District, Jingkai District, and Quanshan District) and five county urban areas (Xinyi, Pizhou, Peixian County, Fengxian County, and Suining County) in Xuzhou City, and 2,871 valid questionnaires were recovered. In the later stage, 194 township health centers (including community hospitals) in Linyi, Shandong, 16 community health service centers in Huaibei, Anhui, and 216 primary public health service organizations in Shangqiu, Henan were selected to carry out more in-depth questionnaire surveys, and 2,840 valid questionnaires were recovered in Henan, 2,910 in Shandong, and 591 in Anhui. 10,665 questionnaires were distributed in the second stage of the survey, and 9,212 questionnaires were eventually recovered. 9,212 questionnaires were finally recovered, with a recovery rate of 86.46%.

### 2.2 Data import and analysis

After the quantitative survey data collection was completed, epidata 3.0 software was used to enter the establishment of the database, and the data were entered twice. After using principal component analysis (PCA) for logical proofreading to deal with unreasonable data and organization, descriptive analysis and processing were carried out using excel and SPSS25.0 statistical analysis software. The baseline characteristics and job satisfaction of grassroots public health personnel were analyzed through descriptive statistical analysis. The relationship between occupational burnout and demographic variables was explored using one-way ANOVA and logistic regression analysis. Additionally, independent samples t-test and one-way ANOVA were employed to investigate the influencing factors of work–family conflict and intention to quit among primary public health personnel.

### 2.3 Baseline characteristics of primary public health care providers

Baseline characteristics of primary public health service workers included age (<35 years, 35–45 years, and > 45 years), sex (male, female), education (specialized and below, bachelor’s degree and above), years of experience (< 5 years, 5–10 years, and > 10 years), hours of work (0–7 h, 8–13 h, 14–21 h, and > 21 h), monthly income [<Ұ3,000 (< $430.9), Ұ3,000–Ұ5,000 ($430.9 to $718.2) , and > Ұ5,000 (>$718.2)], technical title (junior title and below, intermediate, associate and above), whether on-call duties are required (yes, no) and whether on staff (yes, no).

### 2.4 Work–family conflict scale

Work–Family Conflict Assessment using the Chinese version of the multidimensional Work–Family Conflict Scale (WFCS) (18). The Work–Family Conflict (WFC) scale comprises 18 items, encompassing two subscales: work-to-family interference (WIF) and family-to-work interference (FIW). WIF refers to the adverse impact of work-related demands and obligations on an individual’s family life, while FIW implies that conflicts arising from personal-family obligations may disrupt one’s work performance. Both subscales include dimensions such as time conflict, stress conflict, and behavioral conflict. A 5-point Likert scale ranging from 1 (strongly disagree) to 5 (strongly agree) was employed to assess work–family conflict (19). In this study, the Cronbach’s alpha coefficient for WFC was found to be 0.892, and the KMO value was 0.863, indicating good reliability and validity of the scale used in this study.

### 2.5 Copenhagen burnout scale

The Copenhagen Burnout Inventory (CBI) (20), comprising 18 items with a range of 19–90, was utilized as an assessment tool to measure burnout across three dimensions: personal burnout, work-related burnout, and client-related burnout. Additionally, the study conducted an examination of construct validity, factor structure through validated factor analysis, and internal consistency (21). The Cronbach’s alpha values for personal burnout, work-related burnout, and client-related burnout in this study were 0.901, 0.892, and 0.932, respectively. After calculation, the KMO value is 0.863, indicating its high validity.

### 2.6 Minnesota satisfaction scale

The Minnesota Satisfaction Questionnaire Short Scale includes three subscales: intrinsic satisfaction, extrinsic satisfaction, and general satisfaction. The project utilized a 5-point scale where 1 = very dissatisfied with this aspect of my job, 2 = dissatisfied with this aspect of my job, 3 = unsure if I am satisfied or dissatisfied with this aspect of my job, 4 = satisfied with this aspect of my job, and 5 = very satisfied the with this aspect of my job (22). The Cronbach’s alpha coefficient for the MSQ in this study was 0.886, indicating high internal consistency reliability. Additionally, the Kaiser-Meyer-Olkin (KMO) measure of sampling adequacy yielded a value of 0.821, suggesting good construct validity and suitability of the scale.

### 2.7 Intent to leave scale

Respondents’ intention to leave was measured using the Chinese version of the Intention to Leave Questionnaire developed by Cammanette and Mobiley (23). It consists of four items: “Thinking about leaving my current organization,” “Thinking about leaving this industry,” “Currently actively looking for a new job,” and “Planning to look for a new job next year.” A 5-point Likert scale was used to assess all these items, ranging from 1 (strongly disagree) to 5 (strongly agree), with higher scores indicating a tendency to resign. 0The Cronbach’s alpha coefficient for turnover intention in this study was determined to be 0.842, indicating high internal consistency reliability. Additionally, the Kaiser-Meyer-Olkin (KMO) value of 0.902 demonstrated excellent sampling adequacy for the scale used, affirming its strong construct validity.

## 3 Results

### 3.1 Baseline characteristics of the participants

The study shows 9,212 primary public health personnel. The research data are shown in Table 1. There is a higher percentage of females among the primary public health personnel (6079, 66.34%), among which the ratio of men and women in Shangqiu is relatively balanced. 70% of primary public health personnel have a junior college degree or below (6449, 70%), and less than 0.2% of primary public health personnel have a doctor degree or above. Most of the primary public health personnel have more than 10 years of working experience (6,356, 70.22%), while only half of the staff in Shangqiu is working for more than 10 years. The age of the staff in each region is mainly more significant than 35 years old (5,446, 59.11%). In contrast, the age structure of the staff in Shangqiu City is younger, with about 60% of the staff under the age of 35 (1733, 61.02%), and there is a difference at the rate of renewal and replenishment of the staff in each region.

**Table 1:** Baseline characteristics of primary public health workers in the municipalities of Huaihai Economic Zone (*N* = 9,212).

Variable	Xuzhou (%)	Linyi (%)	HuaiBei (%)	ShangQiu (%)
Gender				
Male	35.51%	32.17%	30.85%	42.25%
Female	65.49%	67.83%	69.15%	57.75%
Age (years)				
≤ 25	8.01%	10.58%	5.08%	13.03%
26–30	10.47%	12.82%	7.29%	23.24%
31–35	11.75%	18.63%	8.98%	24.65%
36–40	9.78%	16.87%	10.17%	13.73%
41–45	16.92%	17.18%	26.61%	11.62%
46–50	22.68%	12.82%	24.58%	10.92%
51–55	15.33%	8.21%	14.24%	1.41%
56–60	3.88%	2.23%	2.37%	0.70%
≥61	1.18%	0.65%	0.68%	0.70%
Education				
Junior college and below	72.89%	66.39%	75.59%	79.22%
undergraduate course	27.01%	33.54%	24.07%	20.77%
Master	0.07%	0.00%	0.17%	0.00%
doctor	0.03%	0.07%	0.17%	0.00%
Years of work				
≤2	6.52%	12.68%	6.61%	16.55%
3–5	9.67%	15.53%	10.85%	16.90%
6–9	10.68%	15.70%	6.78%	17.96%
≥10	73.14%	56.08%	75.76%	48.59%

### 3.2 Burnout of the participants

A one-way analysis of variance (ANOVA) was performed to examine the basic characteristics of primary public health workers, as presented in Table 2.

**Table 2:** Single-factor analysis of burnout among primary public health workers.

Variant	*n*	PB			WB			CB			Totals		
		Score	F/t	*p*	Score	F/t	*p*	Score	F/t	*p*	Score	F/t	*p*
(a person’s) age													
<35 years	3,766	19.54 ± 4.634	1.213	0.306	20.87 ± 5.036	1.923	0.162	16.72 ± 4.628	0.613	0.621	57.14 ± 12.964	0.164	0.862
35–45 years	3,091	20.62 ± 4.782			20.41 ± 5.011			16.36 ± 5.013			56.52 ± 13.431		
>45 years old	2,355	21.83 ± 4.936			19.39 ± 4.896			17.11 ± 4.961			57.93 ± 13.162		
Educational attainment													
Specialized and below	6,449	19.73 ± 4.627	−0.928	0.315	20.45 ± 5.023	−0.267	0.742	16.66 ± 4.725	0.524	0.592	56.93 ± 13.012	−0.213	0.796
Undergraduate and above	2,763	20.03 ± 4.741			20.76 ± 5.128			16.49 ± 4.806			57.24 ± 13.317		
Years of experience													
<5 years	1,474	18.64 ± 4.168	11.832	0.000**	20.02 ± 5.024	4.428	0.014*	15.96 ± 4.762	1.504	0.223	54.73 ± 13.098	5.128	0.006*
5–10 years	1,382	20.53 ± 4.742			21.13 ± 5.082			16.87 ± 4.671			58.32 ± 13.216		
>10 years	6,356	20.12 ± 4.523			19.47 ± 5.103			16.72 ± 5.264			56.91 ± 13.728		
Title													
Junior and below	5,950	19.71 ± 4.635	2.245	0.107	20.42 ± 5.147	2.492	0.079	16.69 ± 4.922	0.596	0.524	56.92 ± 14.296	0.324	0.725
Middle level (in a hierarchy)	2,718	20.24 ± 4.512			20.14 ± 4.802			16.51 ± 4.967			57.21 ± 12.816		
Associate senior and above	544	20.53 ± 4.056			18.62 ± 4.532			15.92 ± 4.414			54.86 ± 12.745		
Distinguishing between the sexes													
A male	3,133	19.32 ± 4.425	−3.197	0.001**	20.24 ± 4.812	−1.628	0.114	16.42 ± 4.692	−1.126	0.218	55.72 ± 12.765	−2.145	0.025*
Females	6,079	20.42 ± 4.617			20.81 ± 5.363			16.83 ± 4.921			57.79 ± 13.426		
Working hours													
0–7 h	92	19.34 ± 4.638	2.443	0.057	19.83 ± 4.703	4.072	0.006*	16.49 ± 4.826	2.026	0.121	55.56 ± 13.091	3.156	0.033*
8–13 h	8,127	19.46 ± 4.566			20.25 ± 5.006			16.22 ± 4.948			55.62 ± 13.147		
14–21 h	441	20.46 ± 4.372			21.08 ± 5.328			16.68 ± 4.762			58.32 ± 12.955		
>21 h.	552	20.63 ± 4.981			21.81 ± 5.318			17.72 ± 4.773			60.02 ± 13.854		
Monthly income (¥)^a^													
<3,000	5,018	19.72 ± 4.632	2.02	0.155	20.76 ± 5.223	7.843	0.000**	16.64 ± 4.802	6.643	0.001**	57.32 ± 13.652	6.018	0.003*
3,000 t-, 000	2,721	20.03 ± 4.718			20.91 ± 5.093			16.96 ± 4.924			57.89 ± 13.316		
>5,000	1,473	19.34 ± 4.244			18.75 ± 4.460			14.98 ± 4.503			53.11 ± 11.754		
Need for duty													
Be	2,902	19.26 ± 4.626	2.278	0.021*	20.03 ± 5.168	1.643	0.121	16.27 ± 4.762	1.461	0.191	55.46 ± 13.143	1.851	0.059
Clogged	6,310	20.32 ± 4.626			20.73 ± 5.054			16.89 ± 4.945			57.75 ± 13.203		
Whether or not on staff													
Be	4,154	20.48 ± 4.382	1.024	0.372	19.92 ± 5.364	0.785	0.432	16.94 ± 5.263	0.752	0.504	57.73 ± 13.864	0.086	0.892
Clogged	5,058	19.52 ± 4.517			20.67 ± 4.649			16.72 ± 4.092			57.34 ± 12.167		

^*^ *p* < 0.05, ^**^ *p* < 0.01.

a 1¥ = US $0.14.

#### 3.2.1 Primary public health workers with different demographic characteristics

The burnout scores of public health workers in different age groups were compared, and the workers in the older age group had higher burnout scores, with a statistically significant difference (*p* < 0.01). The comparisons of personal burnout and job burnout showed statistically significant differences (*p* < 0.05). The burnout scores of public health workers of different genders were compared, and there was a statistically significant difference (*p* < 0.05). The female workers had higher burnout scores, with a statistically significant difference (*p* < 0.01).

#### 3.2.2 Primary public health workers with different occupational status

The burnout scores of public health workers with different years of work experience were compared, and the workers with 5–10 years of work experience had higher burnout scores, with a statistically significant difference (*p* < 0.01). The comparisons of personal burnout and job burnout showed statistically significant differences (*p* < 0.01). The burnout scores of public health workers with different monthly incomes were compared, and there was a statistically significant difference (*p* < 0.01). The comparisons of job burnout and patient burnout showed statistically significant differences (*p* < 0.01). The personal burnout scores of public health workers with different duty schedules were compared, and the workers who needed to work on duty had higher burnout scores, with a statistically significant difference (*p* < 0.05). The level of occupational burnout among frontline public health workers was compared based on their daily work hours. The longer the work hours, the higher the score of occupational burnout, and the difference was statistically significant (*p* < 0.05). The difference in job burnout was also statistically significant (*p* < 0.01).

On the basis of conducting a one-way analysis of variance (ANOVA), with occupational burnout total score as the dependent variable, significant variables (including gender, years of work experience, night shift duty, job title, working hours and monthly income) were selected for logistic regression analysis. The results showed that the length of working hours and monthly income level were the effects of the occurrence of high levels of burnout (*p* < 0.05), as shown in Table 3.

**Table 3:** Multifactorial analysis of burnout among primary public health workers.

variant	B	SE	Wald	*p*	95% CI
Gender (female as reference)					
Male	−0.174	0.256	0.464	0.496	(0.508, 1.388)
Years of work experience (based on >10 years as a reference)					
<5 years	0.258	0.347	0.554	0.457	(0.656, 2.554)
5–10 years	−0.159	0.253	0.396	0.529	(0.520, 1.400)
Whether on night duty (take yes as a reference)					
Not have	−0.298	0.236	1.605	0.205	(0.468, 1.177)
Title (associate degree and above as reference)					
Division level and below	−0.711	0.437	2.646	0.104	(0.209, 1.157)
Middle level (in a hierarchy)	−0.503	0.469	1.152	0.283	(0.241, 1.516)
Hours of work (based on >21 h as a reference)					
0–7 h	0.595	0.337	3.11	0.078	(0.936, 3.510)
8–13 h	0.698	0.326	4.591	0.032	(1.061, 3.802)
14–21 h	0.442	0.332	1.773	0.183	(0.812, 2.983)
Monthly income (based on >5,000 (¥^a^)/month as a reference)					
<3,000	−0.672	0.297	5.111	0.024	(0.285, 0.914)
3,000–5,000	−0.477	0.245	3.801	0.051	(0.384, 1.003)

### 3.3 Occupational satisfaction of the participants

For career satisfaction, the analysis of 22 entries yielded a mean score of (72.55 ± 14.33) for primary public health workers, which indicates that most of the primary public health workers are satisfied with their careers at a level above the normal level, but there are still cases of low career satisfaction. Primary public health workers were generally satisfied with the opportunity to be praised and recognized for their work at work (3.89 ± 0.77), while they were dissatisfied with their satisfaction with their salary and benefits situation (2.73 ± 1.05), busyness at work (2.91 ± 0.97), opportunity for promotion in position (3.02 ± 0.83), and performance appraisal system (3.04 ± 0.96).

### 3.4 Work–family conflicts of the participants

The mean score of work–family conflict among primary public health workers was (44.06 ± 13.36), indicating a relatively high level of conflict.

There was no statistically significant difference between the scores for males (44.52 ± 12.92) and females (43.79 ± 13.46). The dimensions of time conflict, behavioral conflict, and stress conflict had scores of (18.82 ± 6.12), (13.67 ± 4.55), and (14.04 ± 4.71) respectively, with time conflict scoring the highest, reflecting pronounced competition for time resources between work and family, likely related to the long working hours of primary public health workers.

The work–family conflict score for those with an undergraduate degree or higher was (42.40 ± 7.26), which was higher than that of other educational groups. The differences in work–family conflict, time conflict, behavioral conflict, and stress conflict scores between different educational levels were all found to be statistically significant (*p* < 0.01).

The intermediate title group scored (41.32 ± 8.28) points and demonstrated higher scores compared to other groups. There was a statistically significant difference in the work–family conflict and stress conflict scores among different job titles (*p* < 0.05), while the time conflict and behavioral conflict did not show statistical significance (*p* > 0.05).

Furthermore, the comparison of work–family conflict and its associated scores in time, behavior, and stress did not yield statistically significant differences for medical personnel of different genders and ages, as shown in Table 4.

**Table 4:** Work–family conflicts among medical personnel of different genders, ages, qualifications and titles.

Sports event	Categorization	Work–family conflict	Scheduling conflict	Behavioral conflicts	Stressful conflict
Distinguishing between the sexes	Male	44.52 ± 12.92	12.82 ± 4.13	10.75 ± 3.96	10.62 ± 4.72
	Female	43.79 ± 13.46	12.76 ± 4.09	11.39 ± 3.62	10.43 ± 4.35
	F	0.172	0.010	3.374	0.286
	*p*	0.659	0.931	0.072	0.634
(a person’s) age	<35 years	45.69 ± 13.40	11.86 ± 4.16	10.23 ± 3.75	10.36 ± 4.24
	35–45 years	42.68 ± 12.04	12.29 ± 4.31	10.86 ± 3.39	10.64 ± 4.37
	>45 years old	39.81 ± 11.71	11.34 ± 3.99	10.65 ± 3.72	10.90 ± 4.36
	F	1.896	1.975	2.298	1.058
	*p*	0.151	0.147	0.102	0.367
Education attainment	Undergraduate and above	44.40 ± 3.76	13.89 ± 3.67	11.28 ± 3.54	11.15 ± 4.24
	Specialized and below	42.14 ± 12.77	12.64 ± 4.65	10.36 ± 3.57	10.07 ± 4.35
	F	9.893	9.046	5.203	7.736
	*p*	0.000	0.000	0.007	0.001
Title	Associate senior and above	39.85 ± 11.36	12.93 ± 3.92	11.48 ± 3.68	10.87 ± 4.34
	Middle level (in a hierarchy)	44.32 ± 13.28	13.30 ± 4.07	11.12 ± 3.78	11.034.67
	Junior and below	41.79 ± 12.56	11.68 ± 4.16	10.53 ± 3.61	10.46 ± 4.25
	F	3.675	2.196	2.014	4.215
	*p*	0.012	0.079	0.113	0.005

### 3.5 Turnover intention of the participants

The mean score of the junior medical staff’s intention to leave was (8.44 ± 3.55). The item with the highest mean score was “Considering leaving the organization you are currently working for,” with a mean score of (2.76 ± 1.32), and the item with the lowest mean score was “Looking for a new job next year,” with a mean score of (1.62 ± 1.03). The theoretical mean value of the scale was 10, indicating a high intention to leave. The lowest mean score was “looking for a new job next year,” with a mean score of (1.62 ± 1.03). The theoretical mean value of the willingness to leave scale is 10, which indicates that the willingness to leave is high, and less than 10 indicates that the overall willingness of the research participants to leave is not high. However, the standard deviation of the willingness to leave is 3.41, indicating that the distribution of the survey respondents’ willingness to leave varies greatly, and it is necessary to analyze further the influencing factors of the willingness to leave.

In order to examine the disparities in Turnover Intention based on various demographic and sociological characteristics, we employed independent samples t-tests or one-way ANOVA with willingness to leave as the dependent variable and gender, age, education, years of work experience, establishment status, and title type as categorical predictors.

In order to analyze the differences in willingness to leave on different demographic and sociological characteristics, we conducted independent samples *t*-test or one-way ANOVA with a willingness to leave as the dependent variable and gender, age, education, years of working experience, establishment status, and type of title as the categorical variables. As a result, employees with different age groups, marital statuses, education, establishment status, job titles, and years of experience have different willingness to leave their jobs, and the differences are statistically significant. There is no statistically significant difference in the willingness to leave the job of survey respondents of different genders, as shown in Table 5.

**Table 5:** Differences in respondents’ turnover intention employment by demographic and sociological characteristics.

Variant	*n*	Score	F/t	*p*
Distinguishing between the sexes				
Male	3,133	8.91 ± 3.402	0.752	0.435
Women	6,079	8.72 ± 3.136		
(a person’s) age				
<35 years	3,766	9.49 ± 3.169	25.237	0.000**
35–45 years	3,091	8.98 ± 3.420		
>45 years old	2,355	8.15 ± 3.099		
Education attainment				
Specialized and below	6,449	8.46 ± 3.274	−5.965	0.000**
Undergraduate and above	2,763	9.53 ± 3.236		
Years of experience				
<5 years	1,474	9.23 ± 3.112	18.136	0.000**
5–10 years	1,382	8.86 ± 3.267		
>10 years	6,356	8.13 ± 3.373		
Whether or not on staff				
Be	4,154	8.87 ± 3.216	4.663	0.0100*
Clogged	5,058	9.15 ± 3.194		
Title				
Associate senior and above	544	8.16 ± 3.103	7.349	0.000**
Middle level (in a hierarchy)	2,718	8.75 ± 3.217		
Junior and below	5,950	8.93 ± 3.308		

^*^ *p* < 0.05, ^**^ *p* < 0.01.

## 4 Discussion

With the implementation of the Healthy China Strategy, primary health care organizations have been given a more prominent role as the foundation of China’s three-tier healthcare service network. However, compared to secondary and tertiary hospitals, primary health care organizations face resource constraints, which poses challenges in managing the ever-increasing workloads (24).

First of all, based on the scoring results of the CBI scale, it is reasonable to judge that most respondents have experienced job burnout. The causes of burnout may be related to long working hours, heavy workloads, low pay received for the work, and insufficient incentives. Therefore, it is necessary to pay attention to other supportive factors and all roles of the society together to help create an excellent working atmosphere (25). The lack of a reasonable growth mechanism and stable funding for primary health care organizations, the long history of difficult working conditions, and the low wages and income have led to a lack of incentive for primary public health workers to work and an intention to leave. The turnover rate of primary health care workers will be high due to their heavy workload, low income, and low sense of achievement. This has a significant impact on the quantity and quality of essential public health services in primary areas (26).

**Figure 1:**
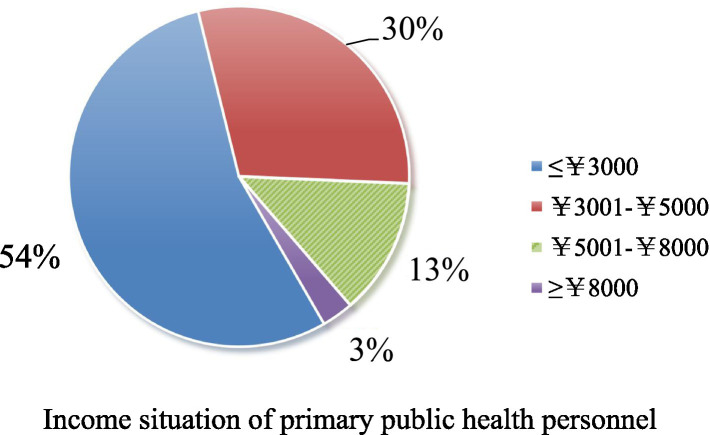
Income situation of primary public health personnel.

**Figure 2:**
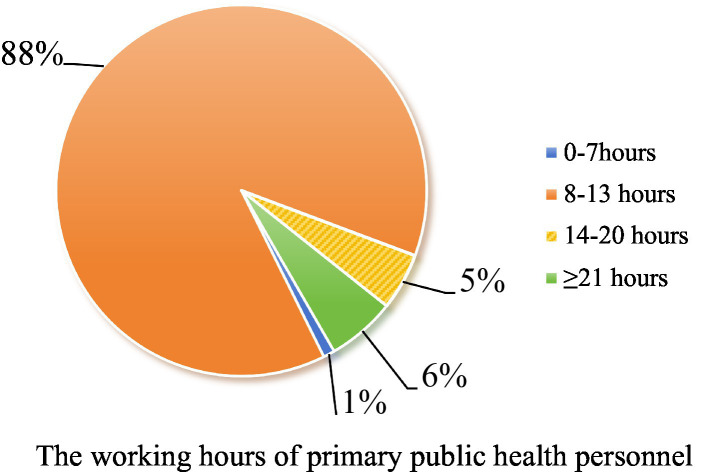
The working hours of primary public health personnel.

Secondly, logistic regression analysis results in this study showed that working hours and monthly income were the influencing factors of high burnout (*p* < 0.05). Regarding the salary level, the cost measurement of the survey sample showed that 54.48% of the income was below 3,000 yuan, and 29.53% was Ұ3,001–Ұ5,000, as shown in Figure 1. This shows that most primary public health workers currently have a low monthly income. Regarding the relationship between income and workload, we found in the survey that 88.31% of primary public health workers work 8–13 h, and the high intensity of workload is difficult for public health workers to bear, as shown in Figure 2. With the continuous increase in the content of public health services at the grassroots level, one person often has to take up several jobs, which are not only very intensive but also very trivial in terms of work content. This, coupled with specific problems in the staffing mechanism of primary public health services, often results in a shortage of workforce (27).

Thirdly, we found the implementation of a comprehensive national health manpower policy at the grassroots level is very much needed (28). Constructing a perfect mechanism to balance the supply and demand of medical personnel training, changing educational concepts, improving training programs, standardizing institutional education, and developing continuing education (29). Grassroots medical and healthcare institutions analyze their own situation and the actual demand for talent, from theoretical training, clinical practice, and intensive training in three aspects: targeted training of practical talents to improve the overall level of medical skills (30). To cultivate a large number of people with the ability to make preliminary judgments on common acute and serious diseases and to master a full range of general medicine and preventive medicine abilities and clinical skills (31). The professionals in the field of general medicine and preventive medicine have been trained to share the pressure and intensity of work in the industry.

The job satisfaction of primary public health workers refers to their degree of recognition and satisfaction with their work, and is a self-assessment of grassroots work (32). It is a self-evaluation of grassroots work. The level of satisfaction affects the efficiency and quality of primary health work and public satisfaction. It has a bearing on primary public health workers’ wastage and physical and mental health. Regarding primary public health workers, most people think they are not skilled enough and can only treat minor illnesses such as colds and fevers. Their impressions of primary public health workers remains that they are “second-rate doctors, “hardly comparable to doctors in large hospitals. As for primary public health workers, they need a higher sense of accomplishment in their work. Of the 9,212 survey samples, only 5,758, or 62.5%, were satisfied with the sense of accomplishment they gained from their work. Relevant studies have proved that primary public health personnel experience a higher sense of security and belonging when they have a higher social status based on the support they receive from their field of work (33). The existing system of medical education, the allocation system of medical students, the grading system of hospitals, the access system of doctors, and other external factors have made it difficult for primary public health personnel to escape from the situation of lower social identity and sense of accomplishment. Therefore, it is imperative to promote the rejuvenation of primary public health workers by introducing new talents and improving benefits (34, 35) to mitigate the loss of skilled professionals. In this regard, leaders of primary health care institutions should respond positively to the opinions and suggestions of their subordinates, establish a relationship of mutual trust with their subordinates, and cultivate a sense of organizational identity and a sense of belonging (36, 37). At the same time, the establishment of a scientific and reasonable compensation system using financial and non-financial incentives, the establishment of a positive organizational culture, mobilization and motivation, thereby increasing their job satisfaction (38).

In the next place, for primary public health workers, chronic work–family conflict serves as a stressor that not only leads to burnout (39), reducing organizational commitment and job satisfaction (40). The work-family balance has a negative impact on the work, and also affects the physical and mental health of medical personnel. Only by maintaining an effective balance between work and family can medical personnel be better engaged in their work (41). For example, the work–family conflict of 35–45 years old primary public health personnel is a major problem. For example, primary public health personnel aged 35–45 have the highest scores of work–family conflict and conflict in all dimensions, probably because the children of personnel in this age group are in the important period of study and growth in junior high school and high school, and because personnel in this age group are in the golden period of their work career, so they feel higher work–family conflict (42). Highly educated primary public health personnel are the backbone or leaders in grassroots work, and their higher commitment to their work exacerbates work–family conflict. Therefore, the alleviation of work–family conflict among primary public health care workers needs to rely on individual efforts and the support of the family and the overall social environment. In addition, hospital administrators should care about the family situation of primary public health personnel, improve their welfare level, strengthen the development of organizational skills, improve work ability, reduce occupational burden, and enhance humanistic care (17, 43).

At the same time, some studies have found that the main factors currently affecting the departure of primary health care workers are low pay, lack of development opportunities and heavy workload. The study showed that due to the limited number of senior titles, the promotion opportunities for primary health care workers are limited, which seriously undermines the willingness of primary health care workers to serve (26). Especially in the health centers of the grassroots townships, the opportunities for further training and learning outside are few and far between. Inadequate hospital management system construction and limited training resources (44). The narrow promotion channels and the lack of career planning (27). These problems have led to the limited ability of primary public health personnel, making it difficult for them to be trusted by the public. The current uneven development of medical and health care will inevitably also have a certain unidirectional orientation of the talent, but also the formation of a vicious circle.

Finally, there is a need to promote digital transformation in the health sector to break down time and space constraints and enhance resource sharing (45). On the one hand, the use of information technology means to realize the “wisdom of health care,” the use of information technology, through distance teaching, online guidance and other means, to open up the barriers to communication between grassroots doctors and specialists, and enrich the training resources to help grassroots doctors to improve their professional level of further training. On the other hand, digital informatization of public health is conducive to reducing the “blind spot” of information inequality, and digital public health is conducive to promoting the exchange and sharing of medical knowledge, experience, and technology; building information systems to meet the needs, coordinating the strength of the information sector, integrating and optimizing the management of hospitals, and inter-operating with the data and information generated by medical care and related behaviors to improve the efficiency of services. The construction of an information system that meets the needs, coordinates the efforts of information departments, integrates and optimizes hospital management, and interoperates data and information generated by medical and related behaviors to improve the service efficiency.

## 5 Limitations

Several limitations should be recognized in this study. First, because the survey was conducted among primary public health workers in four cities in China, Xuzhou, Linyi, Huaibei, and Shangqiu, it has limited generalizability to other populations. Second, the selection bias cannot be excluded because the phase II survey was conducted online, and the results may have self-selection bias. For example, in online surveys, sample selection is not entirely random, and younger junior healthcare workers are more likely to participate in the survey, while older or less proficient in internet use junior healthcare workers may be excluded from the study, leading to insufficient representation of the sample. Finally, this study did not differentiate between different service programs, and future research may find differences in investigating this question in certain profiles of primary public health care workers.

## 6 Conclusion

The following conclusions were drawn from this study: first, the length of work and the level of monthly income were the main factors for the occurrence of high levels of burnout among primary public health workers. Second, most primary public health workers were satisfied with their careers at above-normal levels but were dissatisfied with their salary and benefits, busyness at work, opportunities for the position advancement, and performance appraisal system. Again, time, behavioral, and stress are risk factors for work–family conflict, and competition between work and family is the most evident and can lead directly to work–family conflict. Finally, primary public health workers’ willingness to leave the job varied across sociological characteristics (age, marital status, education, establishment status, job title, and years of service).

The problems junior public health workers face in the Huaihai Economic Zone threaten accessibility, fairness, and people’s health and are not conducive to the promotion of healthcare system reform. Based on the analysis of the current research status and the healthcare policies of Australia and Vietnam (46, 47), this study proposes that countries should strengthen their support for healthcare personnel policies, change training mechanisms based on market demand, address shortages of personnel in primary medical institutions, and alleviate the pressure and intensity of work in the industry. To address issues such as poor social identity and low job satisfaction among primary public health workers, measures should be taken to motivate staff from both spiritual and material perspectives. In this regard, the German government provides additional financial subsidies to attract more doctors to serve rural areas. At the same time, medical digital technology can be utilized to establish a multi-level integrated medical service system like the pyramid-shaped National Health Service (NHS) structure in England which is divided into primary care, secondary care, and tertiary care. General practitioners undertake most primary healthcare work at the bottom of this pyramid structure while referring patients with needs beyond their own capabilities upwards thereby breaking down imbalances in distribution of medical resources while improving resource allocation efficiency.

Currently, there is not enough research on the training of primary health care staff. The research viewpoint is narrow, often based on a single perspective. The research methods are not robust, and much of the relevant research consists of empirical statements and analysis, lacking empirical research (48).

This study conducts an empirical investigation with a large sample size to gain in-depth understanding of the actual working conditions of primary public health personnel, and explores and researches the core issues regarding the development difficulties faced by primary healthcare workers in basic public health services in China from multiple perspectives.

We suggest increasing talent cultivation and recruitment based on the opinions of major administrative departments and international experience, conducting targeted training programs and talent recruitment plans, as well as continuing education to improve employees’ professional service levels. We propose optimizing talent incentive mechanisms to enhance job satisfaction, while strengthening feedback and diverse evaluations to enhance healthcare professionals’ professional identity and improving performance evaluation systems. Measures such as on-the-job internships, case studies, and simulated exercises are recommended to strengthen the practical skills development of talents. Utilizing big data platforms can strengthen information technology construction and promote interdisciplinary integration in order to optimize the path of talent development.

In terms of individual health, research is beneficial for strengthening information communication, reducing the asymmetry of medical information between doctors and patients, and improving the government’s ability to respond to public health emergencies, thereby enhancing residents’ health levels. At the national service system level, research provides a scientific and rational decision-making basis for promoting the formulation and implementation of Huaihai Economic Zone and international public health policies, promoting the integration of medical treatment and prevention to optimize service models, constructing ways to improve service quality and efficiency, as well as strengthening primary healthcare personnel training. It demonstrates certain innovation and cutting-edge qualities.

## Data Availability

The raw data supporting the conclusions of this article will be made available by the authors, without undue reservation.
