# Supplementary material for: Optimization path of primary public health service talent team construction: a largescale survey in Huaihai Economic Zone, China
Source: Front Public Health. 2024 Aug 21;12:1399857. doi: 10.3389/fpubh.2024.1399857 (PMC11371681; doi:10.3389/fpubh.2024.1399857)
Supplement: Supplementary file 1 [file Data_Sheet_1.docx]

# Supplementary Material

**Research related to the construction of national primary public health service talent team**

Dear primary medical staff,

Hello! In order to understand the current status and problems of community-based primary public health services, and to further improve the development of community-based basic public health services in China, we have designed a questionnaire to conduct a survey. This questionnaire is anonymous. You do not need to have any concerns, and we will keep the contents of your answers confidential. Please fill in all the items in the questionnaire truthfully. We would like to express our heartfelt thanks for the support and help you have given us!

"The intensity of the work is scored by the survey respondent on a scale of 0 minimum, 5 medium intensity, and 10 maximum, choosing any score between 0 and 10."

* Please fill in the provincial city and district: [Fill in the blanks] *

_________________________________

*Hospital name: [Fill in the blanks] *

_________________________________

*Your gender: [Single choicee] *

◯ Male ◯ Female

*Your age group: [Single choicee] *

◯25 years old and under

◯26~30 years old

◯31~35 years

◯36~40 years

◯41 to 45 years

◯46 to 50 years

◯51 to 55 years

◯56 to 60 years

◯ 61 years and over

* Your level of education: [Single choicee] *

◯Secondary Schools

◯Tertiary education

◯ Bachelor's degree

◯ Master's degree

◯Doctor

*Category of your specialization: [Single choicee] *

◯ Medical (Western medicine)

◯ Chinese Medicine

◯ Medical technology

◯ Public Health

◯ Care

◯ Pharmacy

◯Other

*You are currently engaged in the position of: [Single choicee] *

◯ Clinical

◯ Medical technology

◯ Public Health

◯ Care

◯ Pharmaceuticals

◯Other

* Your professional and technical title: [Single choicee] *

○No title

○Primary title

○Middle title

○Vice-senior title and above

* Your average monthly income: [Single choicee] *

◯$3,000 and below

◯$3001~$5000

◯$5001~$8000

◯$8001 and above

* Are you currently in an executive business management position: [Single choicee] *

◯ Yes ◯ No

*Are you on staff: [Single choicee] *

◯ Yes ◯ No

*Your years of service: [Single choicee] *

◯ 2 years and under

◯ 3-5 years

◯6-9 years

◯ 10 years and over

*In the past year, has your job required you to work night shifts: [Single choicee] *

◯ Yes (average days/month) ◯ No

* You work about __ hours a day (max 24) [Fill in the blanks] *

**Minnesota Satisfaction Scale**

Below you can find some statements about your current job. Read these statements carefully to determine if you are satisfied with some aspect of your current job as described in the sentences. Then select the option that matches your level of satisfaction.

* Are you satisfied with keeping busy all the time: [Single choicee] *

◯ Very dissatisfied!

◯ Less satisfactory

◯ General

◯ More satisfactory

◯ very satisfied!

*You are interested in working independently: [Single choicee] *

◯ Very dissatisfied!

◯ Less satisfactory

◯ General

◯ More satisfactory

◯ very satisfied!

*You think your work is not monotonous: [Single choicee] *

◯ Very dissatisfied!

◯ Less satisfactory

◯ General

◯ More satisfactory

◯ very satisfied!

* You have the opportunity to be a key player in your team: [Single choicee] *

◯ Very dissatisfied!

◯ Less satisfactory

◯ General

◯ More satisfactory

◯ very satisfied!

*The way you feel about the way your leader treats his subordinates: [Single choicee] *

◯ Very dissatisfied!

◯ Less satisfactory

◯ General

◯ More satisfactory

◯ very satisfied!

* Your ability to make decisions about your leaders: [Single choicee] *

◯ Very dissatisfied!

◯ Less satisfactory

◯ General

◯ More satisfactory

◯ very satisfied!

*You can keep from doing things in your work that are against your conscience: [Single choicee] *

◯ Very dissatisfied!

◯ Less satisfactory

◯ General

◯ More satisfactory

◯ Very satisfied!

*You have stability in your work: [Single choicee] *

◯ Very dissatisfied!

◯ Less satisfactory

◯ General

◯ More satisfactory

◯ very satisfied!

*You are interested in the opportunities this job offers to help others: [Single choicee] *

◯ Very dissatisfied!

◯ Less satisfactory

◯ General

◯ More satisfactory

◯ very satisfied!

*You're interested in the opportunities you have to tell others what to do: [Single choicee] *

◯ Very dissatisfied!

◯ Less satisfactory

◯ General

◯ More satisfactory

◯ Very satisfied!

* You are interested in opportunities to utilize your abilities to the fullest: [Single choicee] *

◯ Very dissatisfied!

◯ Less satisfactory

◯ General

◯ More satisfactory

◯ very satisfied!

* The way you implement hospital policies: [Single choicee] *

◯ Very dissatisfied!

◯ Less satisfactory

◯ General

◯ More satisfactory

◯ very satisfied!

* Your perception of the relationship between your income and your workload: [Single choicee] *

◯ Very dissatisfied!

◯ Less satisfactory

◯ General

◯ More satisfactory

◯ very satisfied!

*You are interested in opportunities for advancement in your position: [Single choicee] *

◯ Very dissatisfied!

◯ Less satisfactory

◯ General

◯ More satisfactory

◯ very satisfied!

*The autonomy of decision-making that you feel you can enjoy in your work: [Single choicee] *

◯ Very dissatisfied!

◯ Less satisfactory

◯ General

◯ More satisfactory

◯ very satisfied!

* Your opportunity to make autonomous decisions about how you want your work done: [Single choicee] *

◯ Very dissatisfied!

◯ Less satisfactory

◯ General

◯ More satisfactory

◯ very satisfied!

*You are interested in the working conditions: [Single choicee] *

◯ Very dissatisfied!

◯ Less satisfactory

◯ General

◯ More satisfactory

◯ very satisfied!

*The way you feel about the way your coworkers get along with each other: [Single choicee] *

◯ Very dissatisfied!

◯ Less satisfactory

◯ General

◯ More satisfactory

◯ very satisfied!

*Rewards you receive for a job well done: [Single choicee] *

◯ Very dissatisfied!

◯ Less satisfactory

◯ General

◯ More satisfactory

◯ very satisfied!

* Your sense of fulfillment in being able to get something out of your work: [Single choicee] *

◯ Very dissatisfied!

◯ Less satisfactory

◯ General

◯ More satisfactory

◯ very satisfied!

* What you learn about organized business training: [Single choicee] *

◯ Very dissatisfied!

◯ Less satisfactory

◯ General

◯ More satisfactory

◯ very satisfied!

*You are interested in the performance appraisal system: [Single choicee] *

◯ Very dissatisfied!

◯ Less satisfactory

◯ General

◯ More satisfactory

◯ very satisfied!

*Your assessment of the overall intensity of current basic public health work: [Single choicee] *

◯ very large

◯ Larger.

◯ General

◯ Smaller.

◯ very small

**Copenhagen Burnout Scale**

*Please answer the following questions based on your feelings and experiences over the past month, and choose the one that best suits your situation. [Matrix Single Choice Question] *

|  | never | minimal | infrequent | non-recurrent | frequent | extremely frequent | Every day. |
| --- | --- | --- | --- | --- | --- | --- | --- |
| 1.You will be exhausted from work | ⭕ | ⭕ | ⭕ | ⭕ | ⭕ | ⭕ | ⭕ |
| 2.Every hour of work makes you feel tired | ⭕ | ⭕ | ⭕ | ⭕ | ⭕ | ⭕ | ⭕ |
| 3.Your work is wearing you out. | ⭕ | ⭕ | ⭕ | ⭕ | ⭕ | ⭕ | ⭕ |
| 4. Your work is emotionally exhausting | ⭕ | ⭕ | ⭕ | ⭕ | ⭕ | ⭕ | ⭕ |
| 5.You have thoughts at work like: I can't take it anymore! | ⭕ | ⭕ | ⭕ | ⭕ | ⭕ | ⭕ | ⭕ |
| 6.You feel weak and may get sick at work | ⭕ | ⭕ | ⭕ | ⭕ | ⭕ | ⭕ | ⭕ |
| 7.You feel weak in the morning when you think of the day's work ahead of you. | ⭕ | ⭕ | ⭕ | ⭕ | ⭕ | ⭕ | ⭕ |
| 8. You feel exhausted at the end of the day. | ⭕ | ⭕ | ⭕ | ⭕ | ⭕ | ⭕ | ⭕ |
| 9.You feel physically exhausted at work | ⭕ | ⭕ | ⭕ | ⭕ | ⭕ | ⭕ | ⭕ |
| 10. your job makes you feel frustrated | ⭕ | ⭕ | ⭕ | ⭕ | ⭕ | ⭕ | ⭕ |
| 11.You are tired of dealing with patients or coworkers at work. | ⭕ | ⭕ | ⭕ | ⭕ | ⭕ | ⭕ | ⭕ |
| 12.You think at work: How long will I be able to work with my patients or colleagues? | ⭕ | ⭕ | ⭕ | ⭕ | ⭕ | ⭕ | ⭕ |
| 13.Working with patients or coworkers drains you of energy | ⭕ | ⭕ | ⭕ | ⭕ | ⭕ | ⭕ | ⭕ |
| 14.You feel tired at work | ⭕ | ⭕ | ⭕ | ⭕ | ⭕ | ⭕ | ⭕ |
| 15.When working with patients or coworkers, you feel that you give more than you get back. | ⭕ | ⭕ | ⭕ | ⭕ | ⭕ | ⭕ | ⭕ |
| 16. You find it difficult to get along and work with patients or coworkers at work | ⭕ | ⭕ | ⭕ | ⭕ | ⭕ | ⭕ | ⭕ |
| 17.you find it frustrating to work with patients or colleagues at work | ⭕ | ⭕ | ⭕ | ⭕ | ⭕ | ⭕ | ⭕ |
| 18.You feel emotionally drained at work | ⭕ | ⭕ | ⭕ | ⭕ | ⭕ | ⭕ | ⭕ |
| 19.You have enough energy to spend with your family and friends in your free time | ⭕ | ⭕ | ⭕ | ⭕ | ⭕ | ⭕ | ⭕ |

**Work-Family Conflict Scale**

The questions in this section are about work-family conflict narratives. Please choose one of the five levels from "Not at all" to "Completely" according to the actual situation of your work. [Matrix Single Choice Question] *

|  | highly disagree | Less disagree | normal | Less agree | highly agree |
| --- | --- | --- | --- | --- | --- |
| 1. My work often prevents me from participating in family activities. | ⭕ | ⭕ | ⭕ | ⭕ | ⭕ |
| 2. I spend too much time at work, which leaves me with insufficient time for family activities | ⭕ | ⭕ | ⭕ | ⭕ | ⭕ |
| 3. I have had to sacrifice time for family activities to accomplish necessary work tasks | ⭕ | ⭕ | ⭕ | ⭕ | ⭕ |
| 4. the time I spend on family obligations usually interferes with the completion of my work | ⭕ | ⭕ | ⭕ | ⭕ | ⭕ |
| 5. Having to spend time with my family prevents me from participating in work activities that would be beneficial to my career. | ⭕ | ⭕ | ⭕ | ⭕ | ⭕ |
| 6. I have to miss work activities because I have to spend a lot of time on family responsibilities | ⭕ | ⭕ | ⭕ | ⭕ | ⭕ |
| 7. when I come home from work, I am often too tired to fulfill my family obligations | ⭕ | ⭕ | ⭕ | ⭕ | ⭕ |
| 8. when I come home from work, I am often depressed and unable to contribute to the household | ⭕ | ⭕ | ⭕ | ⭕ | ⭕ |
| 9. due to pressure at work, sometimes when I get home I can't do my favorite things because I'm too stressed out | ⭕ | ⭕ | ⭕ | ⭕ | ⭕ |
| 10. Due to family stress, I often work while still preoccupied with family matters | ⭕ | ⭕ | ⭕ | ⭕ | ⭕ |
| 11. It is difficult for me to concentrate on my work because of the stress caused by my family responsibilities | ⭕ | ⭕ | ⭕ | ⭕ | ⭕ |
| 12. Tension and anxiety from family life often reduce my ability to work | ⭕ | ⭕ | ⭕ | ⭕ | ⭕ |
| 13. Problem-solving methods used at work are not effective in solving family problems | ⭕ | ⭕ | ⭕ | ⭕ | ⭕ |
| 14. Behaviors that are necessary and effective at work are counterproductive at home | ⭕ | ⭕ | ⭕ | ⭕ | ⭕ |
| 15. effective means of handling work matters do not help me to be a good parent or partner | ⭕ | ⭕ | ⭕ | ⭕ | ⭕ |
| 16. Problem-solving methods used at home are not effective in solving problems at work | ⭕ | ⭕ | ⭕ | ⭕ | ⭕ |
| 17. Behaviors that are necessary and effective at home are counterproductive for work | ⭕ | ⭕ | ⭕ | ⭕ | ⭕ |
| 18. Behaviors that are useful in solving problems in the family do not seem to work effectively at work. | ⭕ | ⭕ | ⭕ | ⭕ | ⭕ |

**Intent to Leave Scale**

*Please choose one of the 5 levels from "Strongly Disagree" to "Strongly Agree" according to the actual situation of your work. [Matrix Single Choice Question] *

|  | very disagree | comparisons  disagree | general | comparisons  agree with | Wholly  agree with |
| --- | --- | --- | --- | --- | --- |
| 1. Thought about leaving current workplace | ⭕ | ⭕ | ⭕ | ⭕ | ⭕ |
| 2. thought about leaving the industry | ⭕ | ⭕ | ⭕ | ⭕ | ⭕ |
| 3. Recently looking for a new job | ⭕ | ⭕ | ⭕ | ⭕ | ⭕ |
| 4. will be looking for a new job next year | ⭕ | ⭕ | ⭕ | ⭕ | ⭕ |
